# Retrospective Analysis of Inpatient Rehabilitation Consultation Referrals and Clinical Profiles in a Tertiary Care Teaching Institute (February 2022 to February 2026)

**DOI:** 10.7759/cureus.111655

**Published:** 2026-06-28

**Authors:** Abhimanyu Vasudeva, Tejas K Patel, Susmita Sarkar, Pratikshya Lenka, Nishat Ahmed Sheikh

**Affiliations:** 1 Physical Medicine and Rehabilitation, All India Institute of Medical Sciences, Gorakhpur, Gorakhpur, IND; 2 Pharmacology and Therapeutics, All India Institute of Medical Sciences, Gorakhpur, Gorakhpur, IND; 3 Prosthetics and Orthotics, All India Institute of Medical Sciences, Gorakhpur, Gorakhpur, IND; 4 Forensic Medicine and Toxicology, All India Institute of Medical Sciences, Deoghar, Deoghar, IND

**Keywords:** inpatient consultation, neurological disorders, physical medicine and rehabilitation, referral patterns, rehabilitation, retrospective study, tertiary care

## Abstract

Background

Rehabilitation is increasingly viewed as a vital part of comprehensive inpatient care, especially in large tertiary hospitals. Despite this growing recognition, there is still limited published information on how inpatient rehabilitation consultations are conducted in newly established tertiary care teaching hospitals in India.

Objectives

The primary objective of this study was to analyze temporal trends in inpatient rehabilitation consultations at All India Institute of Medical Sciences (AIIMS) Gorakhpur, Gorakhpur, Uttar Pradesh, India. The secondary objective was to describe the demographic characteristics of referred patients, the referring clinical departments, and the diagnostic categories prompting rehabilitation consultation.

Methods

A retrospective, observational, record-based study was conducted using inpatient rehabilitation consultation records maintained by the Department of Physical Medicine and Rehabilitation at AIIMS Gorakhpur from February 2022 to February 2026. Data on referral trends, referring departments, patient demographics (age and sex), and primary diagnostic categories were extracted. Records were screened using predefined eligibility criteria, and duplicate and incomplete records were excluded. Descriptive statistics were used for analysis. Ethical approval was obtained from the Institutional Human Ethics Committee (IHEC/AIIMS-GKP/BMR/683/2026).

Results

A total of 1,025 inpatient rehabilitation consultations were included in the analysis. The number of consultations increased during the study period, rising from 61 in 2022 to 418 in 2025. The Department of Medicine was the leading source of referrals (52.3%), followed by Pulmonary Medicine (12.2%), Other departments (12.1%), Pediatrics (10.5%), Dermatology (6.6%), and the Department of Surgery (5.5%). The median age of referred patients was 60 years (IQR, 40-70), and 56.9% were male. Neurological conditions were the most frequent indication for rehabilitation consultation, accounting for 35.7% of all referrals.

Conclusion

The number of inpatient rehabilitation consultations increased during the study period. Most referrals originated from medical specialties and involved older adults with neurological or other complex medical conditions. These findings provide useful information for rehabilitation service planning, workforce development, and resource allocation in tertiary care teaching hospitals. The findings should be interpreted in light of the retrospective single-center design and the inherent limitations of routinely collected clinical data.

## Introduction

Physical medicine and rehabilitation (PMR) aims to help individuals achieve the highest possible level of function and independence, rather than focusing solely on a specific illness or body system. Physiatrists collaborate with multidisciplinary teams, using therapies, medical interventions, and assistive measures, to provide holistic, patient-centered care [1]. As healthcare continues to evolve, PMR has grown from a specialty primarily focused on managing musculoskeletal and neurological impairments, trauma recovery, and disability-related conditions to a vital component of comprehensive, patient-centered care. Beyond its traditional role in restoring function and improving quality of life, PMR now plays an increasingly important part in integrative, preventive, and multidisciplinary healthcare. Rehabilitation specialists contribute across the continuum of care by addressing the interconnected challenges of acute illnesses, chronic diseases, long-term disabilities, and broader population health needs, reflecting the expanding scope and significance of the specialty in modern healthcare systems [2].

In tertiary care teaching hospitals, inpatient rehabilitation consultation patterns may reflect institutional growth, interdisciplinary collaboration, disease burden, and evolving awareness of the role of rehabilitation in acute care settings. Early rehabilitation involvement has been associated with improved functional recovery, prevention of hospital-associated deconditioning, reduction in complications related to immobility, and facilitation of discharge planning [3].

Despite the expanding role of inpatient rehabilitation services, there remains limited literature from emerging tertiary care institutes in India evaluating inpatient rehabilitation consultation trends and referral patterns. Most available Indian rehabilitation literature focuses on specialty-specific rehabilitation outcomes rather than hospital-wide inpatient rehabilitation consultation patterns [4].

Understanding referral trends, demographic characteristics, and broad diagnostic categories requiring rehabilitation consultation may help identify evolving institutional needs, interdisciplinary referral patterns, and future service development priorities.

The primary objective of this study was to analyze temporal trends in inpatient rehabilitation consultations at All India Institute of Medical Sciences (AIIMS) Gorakhpur, Gorakhpur, Uttar Pradesh, India, from February 2022 to February 2026. The secondary objective was to describe the demographic characteristics of referred patients, the referring clinical departments, and the primary diagnostic categories prompting rehabilitation consultation.

## Materials and methods

Study design

This was a retrospective, observational, record-based descriptive study conducted in the Department of PMR at AIIMS Gorakhpur.

Data source

Data were obtained from the department’s inpatient rehabilitation consultation register maintained as part of routine clinical documentation. The register included details of admitted inpatients referred from various clinical departments for rehabilitation consultation during their hospital stay. These referrals represented inpatient rehabilitation consultations in admitted ward patients and did not represent transfers to rehabilitation units or outpatient referrals.

Study duration

The study included all eligible inpatient rehabilitation consultation records documented between February 2022 and February 2026.

Study population

A total of 1,100 consultation records were screened using predefined eligibility criteria.

Inclusion criteria

All documented inpatient rehabilitation consultation entries recorded in the departmental register during the study period were included.

Exclusion criteria

Records with missing essential study variables required for analysis, records containing invalid date entries, and duplicate consultation records were excluded. During final manual verification, records with multiple irretrievable missing essential variables were also excluded.

Variables collected

The variables extracted included year of referral, referring clinical department, age, sex, and the principal diagnostic category prompting rehabilitation consultation. Diagnostic categories were assigned using predefined operational definitions based on the principal diagnosis prompting rehabilitation consultation. Neurological conditions included disorders affecting the central or peripheral nervous system. Cardiopulmonary conditions included cardiovascular and respiratory disorders requiring rehabilitation interventions. Wound/dermatological conditions included pressure injuries, wounds, burns, and other dermatological disorders requiring rehabilitation. Post-operative/surgical conditions included patients referred following surgical procedures requiring rehabilitation. Musculoskeletal/rheumatologic conditions included disorders affecting bones, joints, muscles, connective tissues, and related structures. Pediatric developmental conditions included developmental disorders requiring rehabilitation. The “Other” category included systemic, metabolic, infectious (including sepsis), and miscellaneous conditions that did not meet the predefined criteria for the other diagnostic categories. Where multiple diagnoses were documented, a single principal diagnosis representing the primary indication for rehabilitation consultation was assigned using predefined classification criteria.

Data handling and confidentiality

All data were anonymized before analysis by removing personal identifiers, including patient names and hospital identification numbers. Data extraction and preprocessing were performed using Microsoft Excel (Microsoft 365 Personal, Microsoft Corporation, Redmond, WA). Microsoft Copilot was used solely to assist in the preliminary identification of potential duplicate records, incomplete entries, and inconsistent variable formats. Because of row-processing limitations, the dataset was divided into smaller Excel worksheets, processed in batches, and subsequently merged.

Duplicate records were defined as repeated consultation entries referring to the same inpatient consultation. Incomplete records were defined as those lacking one or more essential study variables required for analysis, including referral date, referring department, age, sex, or the principal diagnostic category. Records with invalid date entries were excluded before data cleaning. During the final manual verification process, three additional records containing multiple irretrievable missing essential variables were identified and excluded.

All artificial intelligence (AI)-assisted outputs were independently reviewed and verified by the investigators. Eligibility assessment, duplicate identification, assignment of the principal diagnostic category, and resolution of discrepancies were performed through structured multi-investigator review and consensus-based adjudication. AI-assisted outputs were used only to facilitate preliminary data screening and did not replace investigator judgment at any stage of data cleaning, classification, or analysis.

Statistical analysis

Data were tabulated and analyzed using descriptive statistics in Microsoft Excel (Microsoft 365 Personal). Categorical variables are presented as frequencies and percentages, while continuous variables are summarized using the median and interquartile range (IQR). Temporal trends were described using annual consultation counts.

Ethics approval

Ethical approval for the study was granted by the Institutional Human Ethics Committee (IHEC/AIIMS-GKP/BMR/683/2026).

## Results

**Figure 1:**
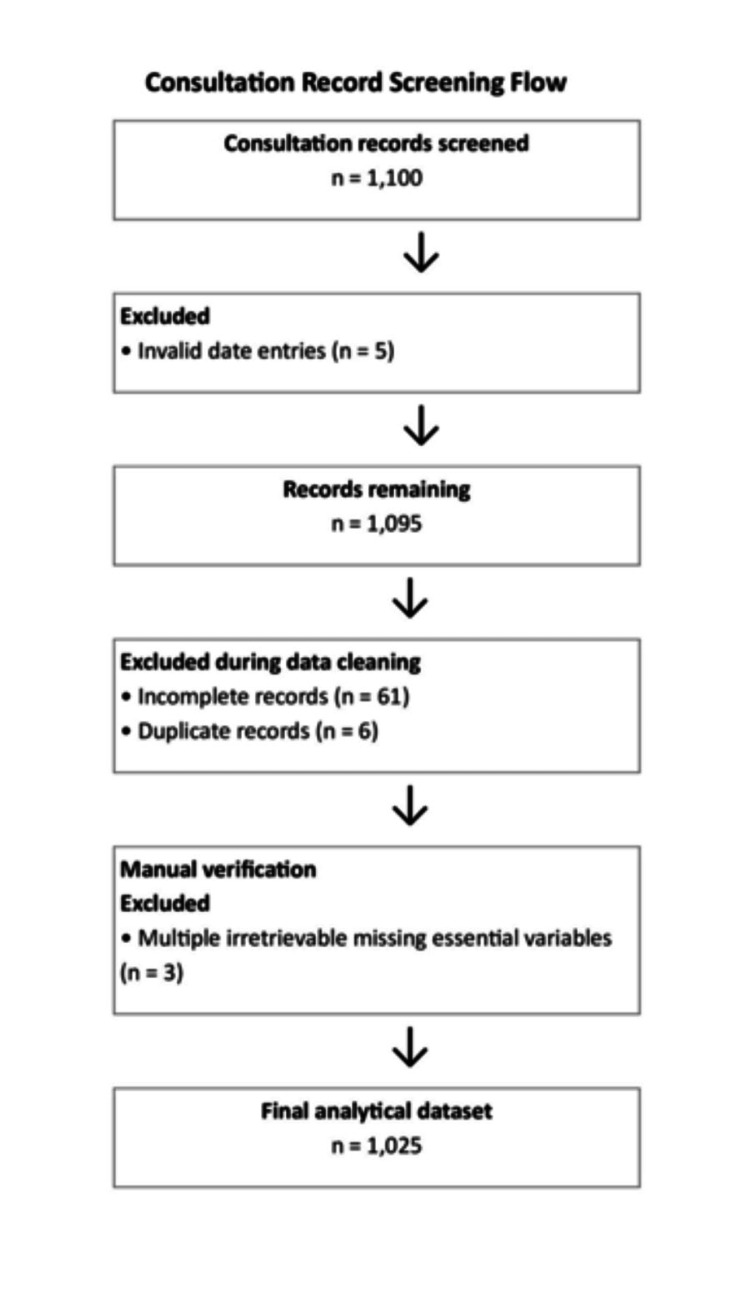
Flow diagram of consultation record screening and inclusion.

A total of 1,100 consultation records were screened. Five records with invalid date entries were excluded. Of the remaining records, 61 incomplete records and six duplicate records were excluded during data cleaning. During final manual verification, three additional records containing multiple irretrievable missing essential variables were excluded. The final analytical dataset comprised 1,025 inpatient rehabilitation consultation records (Figure 1).

The annual number of inpatient rehabilitation consultations increased during the study period, from 61 consultations in 2022 to 418 in 2025. Data for 2022 and 2026 represent partial years (February to December 2022 and January to February 2026, respectively). Year-wise consultation counts are presented in Table 1.

**Table 1:** Annual inpatient rehabilitation consultation counts (February 2022 to February 2026). *2022 includes data from February to December, and 2026 includes data from January to February.

Year	Number of Consultations
2022*	61
2023	181
2024	314
2025	418
2026*	51

**Figure 2:**
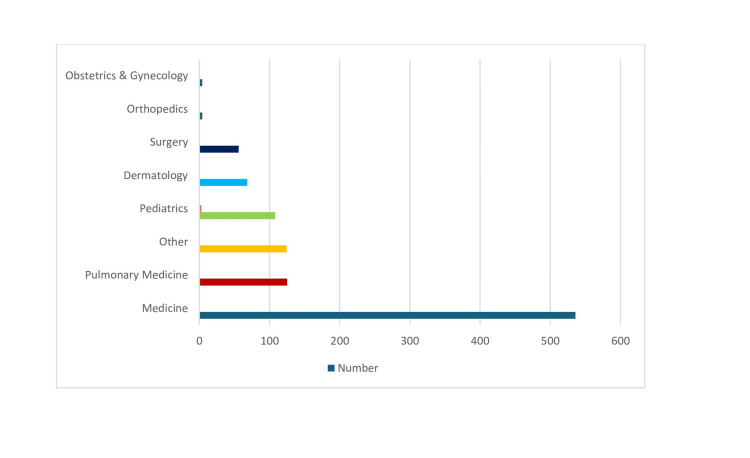
Distribution of inpatient rehabilitation consultations according to the referring clinical department.

The Department of Medicine was the leading source of referrals (536, 52.3%), followed by Pulmonary Medicine (125, 12.2%), other clinical departments (124, 12.1%), Pediatrics (108, 10.5%), Dermatology (68, 6.6%), and the Department of Surgery (56, 5.5%). Orthopedics and Obstetrics and Gynecology contributed a small proportion of referrals (4, 0.4% each) (Table 2, Figure 2).

**Table 2:** Distribution of referring clinical departments. *Other comprises referrals from the remaining clinical departments not presented individually in the table.

Department	Number (%)
Department of Medicine	536 (52.3%)
Pulmonary Medicine	125 (12.2%)
Other*	124 (12.1%)
Pediatrics	108 (10.5%)
Dermatology	68 (6.6%)
Department of Surgery	56 (5.5%)
Orthopedics	4 (0.4%)
Obstetrics and Gynecology	4 (0.4%)
Total	1,025 (100%)

The median age of the cohort was 60 years (IQR 40-70). Of the 1,025 patients, 583 (56.9%) were male and 442 (43.1%) were female. Patients aged ≥60 years accounted for approximately 47% of the study population. Pediatric patients accounted for approximately 7% of the study population (Table 3).

**Table 3:** Age distribution.

Age Group	Number (%)
<1 year	16 (1.6%)
1–4 years	19 (1.9%)
5–11 years	21 (2.1%)
12–17 years	18 (1.8%)
18–39 years	210 (20.5%)
40–59 years	261 (25.5%)
60–74 years	325 (31.7%)
≥75 years	155 (15.1%)

Neurological conditions were the most common principal diagnostic category (35.7%), followed by the "Other" category (31.1%), cardiopulmonary conditions (17.8%), wound/dermatological disorders (6.4%), post-operative/surgical conditions (3.9%), musculoskeletal/rheumatologic disorders (3.6%), and pediatric developmental disorders (1.5%) (Table 4).

**Table 4:** Broad diagnostic categories prompting rehabilitation consultation. *The "Other" category includes systemic, metabolic, infectious (including sepsis), and miscellaneous conditions that did not meet the predefined criteria for the other diagnostic categories.

Diagnostic Category	Number (%)
Neurological	366 (35.7%)
Cardiopulmonary	182 (17.8%)
Other*	319 (31.1%)
Wound/dermatological	66 (6.4%)
Post-operative/surgical	40 (3.9%)
Musculoskeletal/rheumatologic	37 (3.6%)
Pediatric developmental	15 (1.5%)

## Discussion

The present study observed a substantial increase in inpatient rehabilitation consultation referrals over the four-year study period at AIIMS Gorakhpur. The progressive rise in referrals may reflect increased institutional integration of rehabilitation services, improved interdisciplinary awareness, and growing recognition of the role of rehabilitation during hospitalization.

The nearly seven-fold increase in consultations between 2022 and 2025 is noteworthy. In developing tertiary care systems, inpatient rehabilitation consultation services often evolve gradually alongside the expansion of inpatient specialty services and the increasing complexity of hospitalized patients. International prospective stroke cohorts have emphasized the importance of rehabilitation as an integral component of long-term post-stroke care pathways [5]. However, the consultation counts for 2022 and 2026 represent partial-year data and should therefore be interpreted accordingly.

The finding that most referrals in the present study originated from the Department of Medicine aligns with existing hospital-based consultation-liaison literature, in which Medicine is consistently reported as the leading source of inpatient referrals [6]. The high proportion of referrals from Pulmonary Medicine may additionally reflect increasing utilization of pulmonary rehabilitation principles, bedside mobilization, and functional conditioning in patients with respiratory illnesses. Pulmonary rehabilitation has attracted increasing attention because of its demonstrated benefits in improving functional capacity, symptom management, and quality of life. Growing interest is now focused not only on the initial rehabilitation phase but also on strategies to sustain these gains over the long term, including maintenance programs, technology-enabled delivery models, and behavior change interventions. Despite this expanding focus, the optimal approach for long-term rehabilitation delivery and support remains an active area of research [7].

Neurological disorders constituted the most common indication for rehabilitation consultation. Stroke and other neurological illnesses are among the leading causes of disability worldwide and frequently require early multidisciplinary rehabilitation intervention [8]. Similar predominance of neurological diagnoses has been reported in hospital-based neurology consultation studies, while rehabilitation literature also reflects a high burden of neurological conditions in inpatient rehabilitation settings [9,10].

The substantial proportion of cardiopulmonary and other systemic illness-related referrals highlights the expanding role of PMR beyond conventional orthopedic or musculoskeletal rehabilitation. Hospitalized patients with chronic respiratory disease, heart failure, sepsis, prolonged immobilization, critical illness, and other systemic conditions commonly develop significant functional impairment requiring rehabilitation assessment and intervention [11,12].

The demographic profile demonstrated a predominance of older adults. Patients aged 60 years or older are particularly vulnerable to hospital-associated deconditioning, sarcopenia, functional decline, and immobility-related complications during hospitalization [3]. Early rehabilitation consultation in such patients may contribute to the maintenance of mobility, prevention of secondary complications, and optimization of discharge outcomes.

Referrals from Orthopedics and from Obstetrics and Gynecology remained comparatively limited. This may reflect evolving interdisciplinary referral pathways, variable awareness regarding rehabilitation indications, or specialty-specific inpatient management patterns. Similar variability in referral utilization between departments has been reported in consultation-liaison literature [6].

The present study has several strengths, including the analysis of a large real-world dataset of inpatient rehabilitation consultations collected over four years, comprehensive institutional consultation capture, and representation of referrals from diverse clinical specialties within an evolving tertiary care teaching hospital. Nevertheless, the study has several limitations. Its retrospective single-center design may limit the generalizability of the findings. The absence of an admissions denominator precluded calculation of referral rates, and data for 2022 and 2026 represented partial years. Diagnostic classification was based on a single principal diagnosis without formal assessment of inter-rater reliability. In addition, the heterogeneous nature of the “Other” diagnostic category may limit the interpretability of diagnostic distributions despite the use of predefined operational definitions. Detailed functional outcome measures, disease severity, and longitudinal follow-up data were unavailable because of the retrospective record-based design.

Nevertheless, the findings provide important insights into the evolving role of inpatient rehabilitation consultation services in tertiary care teaching hospitals and may help inform service planning, resource allocation, workforce planning, and future multicenter studies.

## Conclusions

This study provides a comprehensive overview of inpatient rehabilitation consultation patterns at AIIMS Gorakhpur over a four-year period. Inpatient rehabilitation consultations increased progressively during the study period, with referrals originating predominantly from the Department of Medicine and neurological disorders constituting the most common indication for rehabilitation consultation. These findings highlight the expanding integration of PMR within acute inpatient care and provide baseline data that may inform service planning, resource allocation, and workforce development. Future multicenter prospective studies incorporating functional outcomes and standardized referral metrics are warranted to further characterize inpatient rehabilitation needs and optimize rehabilitation service delivery.
